# γ‑Graphyne as a Functional 2D Nanoarchitectonics for Room-Temperature Chemiresistive–Potentiometric Sensing Interfaces

**DOI:** 10.1021/acssensors.5c01507

**Published:** 2025-10-02

**Authors:** Utkarsh Kumar, Pei-Ying Wu, Chun-En Lin, Zu-Yin Deng, Ren-Jang Wu, Kuen-Lin Chen, Wen-Min Huang, Chiu-Hsien Wu

**Affiliations:** † Department of Physics, 34916 National Chung Hsing University, Taichung 402, Taiwan; ‡ I - Center for Advanced Science and Technology (I CAST), National Chung Hsing University, Taichung 402, Taiwan; § Institute of Nanoscience, 34916 National Chung Hsing University, Taichung 402, Taiwan; ∥ Department of Applied Chemistry, 63293 Providence University, Taichung 43301, Taiwan

**Keywords:** graphyne, gas sensing, 2D material, DFT, deep analysis

## Abstract

The development of highly selective gas sensors operating at room temperature with detection capabilities in the parts-per-billion (ppb) range is one of fundamental and technological interest across diverse fields. Conventional sensor arrays often suffer from signal instability, large device footprints, and high fabrication costs. The emergence of two-dimensional (2D) materials has enabled new paradigms in chemiresistive sensing, leveraging their quantum confinement, high surface-to-volume ratio, and tunable electronic structure. In this study, we present, for the first time, a high-performance chemiresistive sensor based on chemically exfoliated γ-graphyne, a carbon allotrope with sp–sp^2^-hybridized bonding and an extended π-conjugated system. The material’s cross-linked layered structure introduces spatially varying local potential gradients, which enhance charge carrier modulation upon gas molecule adsorption. First-principles density functional theory (DFT) calculations were employed to optimize the graphyne synthesis pathway and to model adsorption energies and charge transfer dynamics. Real-time detection of NO_2_ gas at room temperature demonstrates exceptional sensor performance, with a measured response of 1.05 at 25 ppb and an estimated detection limit as low as 0.45 ppb. The device exhibits rapid response (53 s) and recovery (185 s) times, governed by gas-adsorbate interactions and carrier scattering mechanisms. Theoretical models reveal that adsorption of NO_2_ induces significant modulation of the local density of states and carrier concentration in graphyne, enhancing its chemiresistive response. Furthermore, we integrated machine learning algorithms with the experimental sensor output to establish a robust gas classification framework. Classifiers trained on sensor data exhibit 100% accuracy across varying concentrations (15–100 ppb) of NO_2_ and high selectivity for other interfering gases, validating the discriminatory power of the sensor. This synergistic approach combining quantum mechanical modeling, charge transport physics, and data-driven learning algorithms opens new avenues for designing next-generation miniaturized gas sensors with ultrahigh sensitivity and selectivity.

## Graphical abstract

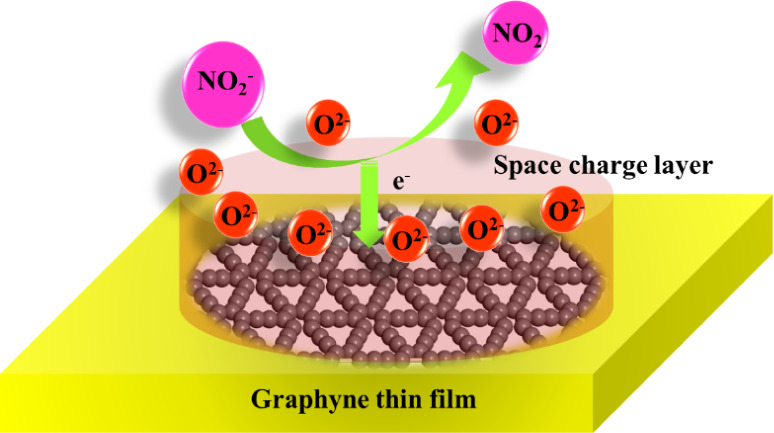

The carbon nanomaterial family has gained significant attention in various fields over the last few decades. Carbon nanomaterials, such as carbon nanotubes (CNTs),^1–3^ graphene,^4,5^ fullerenes,^6^ and nanodiamonds,^7^ hold significant importance across various fields due to their exceptional properties. Carbon nanoarchitectonics,^8^ especially graphene and CNTs, exhibit high electrical conductivity, making them ideal for transistors, conductive films, and interconnects in electronic circuits.^9^ Their nanoscale dimensions allow for the development of smaller and faster electronic components, which are crucial for advancing microelectronics, quantum computing, and electrochemical sensors.^10–13^ Carbon nanomaterials are utilized in electrodes for batteries (e.g., lithium-ion batteries) and supercapacitors due to their high surface area, conductivity, and ability to facilitate rapid charge/discharge cycles.^2,14^ They are also employed as catalyst supports in fuel cells, enhancing the efficiency and stability of catalytic processes.^15^ The high surface area and sensitivity of carbon nanomaterials enable the detection of chemical and biological substances at extremely low concentrations.

They are employed in sensors to detect pollutants, toxic gases, and heavy metals in the environment. Carbon nanomaterials can be functionalized to carry drugs and target specific cells or tissues, enhancing the efficacy and reducing the side effects of treatments.^16^ They are used in imaging techniques, such as MRI and fluorescence imaging, due to their unique optical and magnetic properties. Carbon nanomaterials create scaffolds that support cell growth and tissue regeneration. They are used in coatings to enhance surface properties, such as hardness, conductivity, and resistance to corrosion and wear. Carbon nanomaterials are used in filtration systems to remove contaminants, heavy metals, and organic pollutants from water. They are employed in air filters to capture pollutants and hazardous gases, improving air quality.^17,18^ Carbon nanomaterials enhance the efficiency of photodetectors and solar cells by improving light absorption and charge transport. Due to their excellent optical properties, they are used in organic light-emitting diodes (OLEDs) and other display technologies.^19,20^

Graphene and graphyne are both carbon-based materials, but they differ significantly in their atomic structure and properties.^21^ Graphene is a single layer of carbon atoms arranged in a hexagonal lattice, known for its exceptional electrical conductivity, mechanical strength, and thermal properties. It consists entirely of sp^2^-hybridized carbon atoms, which results in a highly delocalized π-electron system across its two-dimensional plane. This structure gives graphene remarkable electronic properties, such as high electron mobility and the quantum Hall effect. On the other hand, graphyne is a lesser-known but intriguing carbon allotrope that also consists of carbon atoms arranged in a two-dimensional lattice.^21^ However, unlike graphene, graphyne includes sp^2^ and sp-hybridized carbon atoms, leading to a structure with alternating triple (acetylenic) and double bonds between carbon atoms. This unique bonding arrangement creates a more diverse range of electronic properties compared to graphene, such as tunable band gaps and anisotropic conductivity, depending on the specific type of graphyne.^22^

The importance of graphyne lies in its potential for applications where graphene’s properties might be limited. For instance, graphyne’s tunable electronic structure makes it a promising candidate for semiconducting materials in nanoelectronics, where a controlled band gap is crucial.^23^ Additionally, its distinctive structure offers enhanced chemical reactivity and selectivity, making it suitable for use in gas sensing, catalysis, and energy storage devices.^24^ The ability to tailor the properties of graphyne by altering its bonding patterns opens up new avenues for the development of advanced materials in fields ranging from electronics to environmental sensing.^25^

## Materials and Methods

Calcium carbonate (CaC_2_ purity ∼82%) and hexabromo benzene (PhBr_3_ purity ∼97%) were purchased from Yuva Trading. Ethanol (purity >99.5%) was purchased from Jungming Chemicals. 1-Methyl-2-pyrrolidone (purity >99%) and nitric acid (purity ∼ 50–70%) were also purchased from Yuva Trading. Gases such as NO_2_ (purity >99.9%), CO (purity >99.9%), and CH_4_ (purity >99.9%) were purchased from Oriental Gases .

### Synthesis of Graphyne

Graphyne was synthesized using a chemical exfoliation method that began with grinding calcium carbide (CaC_2_) powder for 12 h to create ultrafine particles. These particles were then mixed with phenol benzene (PhBr_6_) in 50 mL of ethanol and stirred for 1 h, during which bromide ions (Br^–^) attached to the carbon layers, disrupted the carbon bonds,^26,27^ This allowed triple bonds to form between carbon atoms, facilitating the assembly of graphyne structures. The schematic evaluation of the structure is depicted in Figure 1. The solution was subsequently ultrasonicated for 24 h to promote the exfoliation of graphyne layers. The exfoliated layers were centrifuged at 6000 rpm for 30 min and washed multiple times with ethanol, *N*-methyl-2-pyrrolidone (NMP), and water to remove impurities, resulting in purified graphyne layers. The schematic reactions for the synthesis of graphyne are given as

1 $$2CaC_{2}+2PhBr_{6}\overset{C_{2}H_{5}OH+Ultrasonication}{\to }PhBr_{4}C_{2}\cdot \cdot \cdot PhBr_{4}C_{2}+2CaBr_{2}↑$$

2 $$2CaC_{2}+PhBr_{4}C_{2}\cdot \cdot \cdot PhBr_{4}C_{2}\overset{C_{2}H_{5}OH+Ultrasonication}{\to }PhBr_{2}C_{4}\cdot \cdot \cdot PhBr_{2}C_{4}+2CaBr_{2}↑$$

3 $$nCaC_{2}+nPhBr_{2}C_{4}\cdot \cdot \cdot PhBr_{2}C_{4}\overset{C_{2}H_{5}OH+Ultrasonication}{\to }Graphyne+nCaBr_{2}↑$$

**Figure 1:**
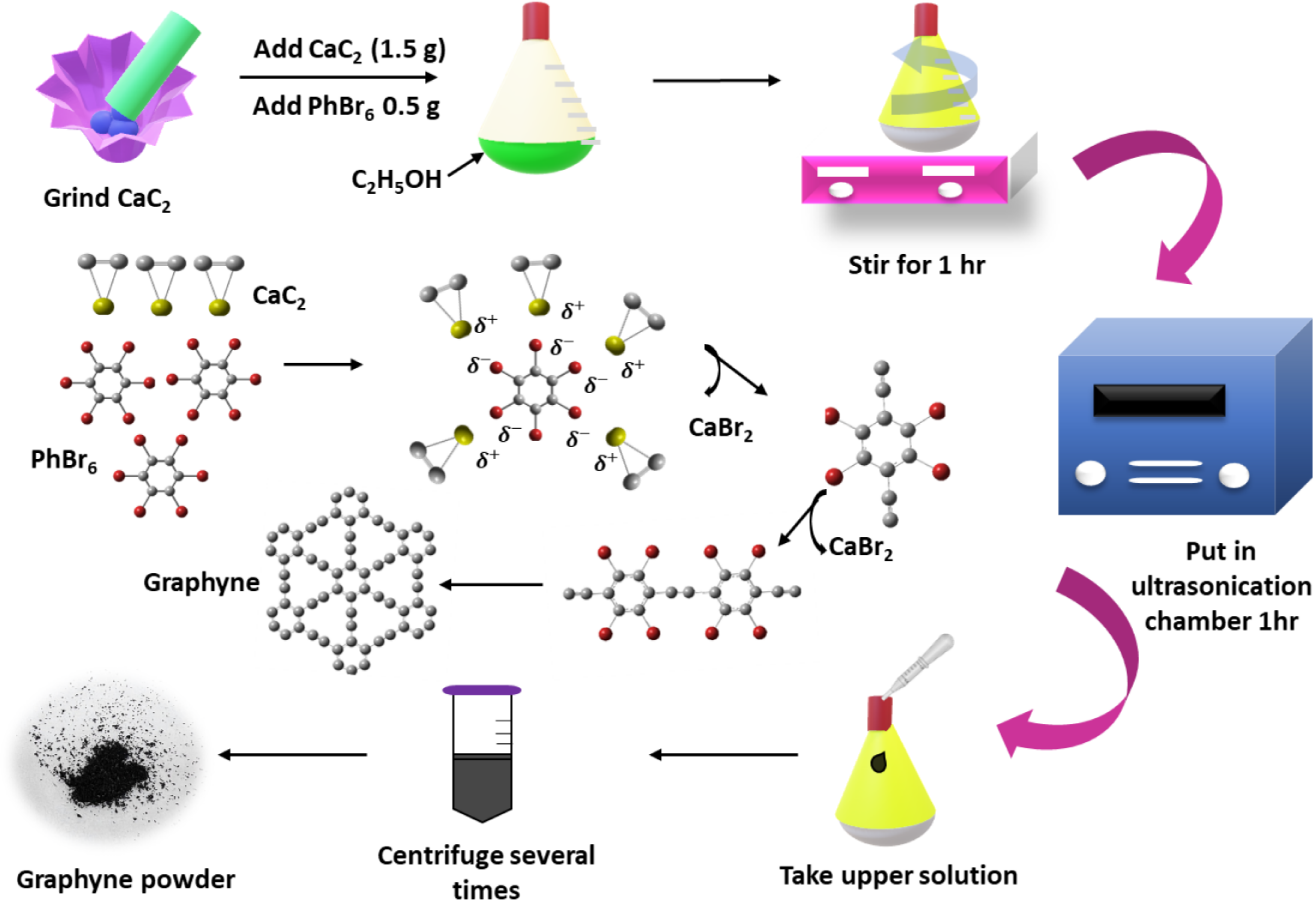
(a) Schematic diagram of graphyne synthesis by using a chemical method.

The reaction mechanism has been validated through computational analysis using Gaussian software,^28^ as depicted in Figure S1. The results indicate a progressive increase in the system’s optimized energy at each reaction step. To facilitate the desired reaction pathway, ultrasonication is employed as an external energy source. Ultrasonic waves impart localized energy at the surface of the reactants, promoting bond dissociation and enhancing molecular interactions. This energy aids in breaking existing bonds and facilitating the recombination of atoms and molecules, thereby driving the formation of graphyne.

### Characterization Technique

Elemental mapping and scanning electron microscopy (SEM) images were obtained by using a field emission scanning electron microscope equipped with an energy-dispersive X-ray spectrometer (EDX). Transmission electron microscopy (TEM) images were taken with a Tecnai G2 microscope operating at an accelerating voltage of 200 kV. X-ray diffraction (XRD) analysis was carried out with a PANalytical B.V. diffractometer (Netherlands), employing CuKα radiation and covering a 2θ range from 10 to 80 degrees. The structural and molecular characterizations were carried out by using advanced analytical instruments. Fourier Transform Infrared (FTIR) spectra were recorded on a PerkinElmer Spectrum 100, Raman spectra were acquired with a NANOSCOPE NS220-RM0121010 Raman spectrometer, and Nuclear Magnetic Resonance (NMR) spectra were obtained by using a JEOL ECZ400S/L1 NMR spectrometer. As described in our previously published work, gas sensing evaluations were conducted using a custom-built gas sensing setup.^29–31^

### Computational Method

Quantum mechanical density functional theory (DFT) calculations were performed using the CASTEP module in Materials Studio software, specifically for modeling the surfaces of electronic structures and the binding properties of carbon allotropes. We employed the generalized gradient approximation (GGA) method, utilizing the nonempirical local functional, Perdew–Burke–Ernzerhof (PBE) correlation, and a double numerical basis plus polarization (DNP) basis set. This approach is well-established in materials science and physics. The calculations were carried out at 0 K, without pressure, and excluded zero-point motion. Machine learning was performed using the MATLAB platform.^32^

## Results and Discussion

After the 2D graphyne was successfully synthesized by using a fast and easy ultrasonication method, the material was characterized by using several advanced techniques, as shown in Figure 2. The HRTEM images of 2D graphyne are illustrated in Figure 2a,b on a scale of 200 nm. The graphyne appears as a network of thin, layered sheets, indicative of its two-dimensional nature. The sheets exhibit varying degrees of transparency, suggesting differences in thickness and possible stacking of layers. In some areas, the sheets overlap, creating regions of higher contrast, which may indicate multilayer formation. The image also shows a few larger, more opaque regions that could correspond to thicker accumulations or agglomerations of graphyne material.^22^ Additionally, the presence of some irregular shapes and boundaries in the material may point to defects or impurities within the graphyne structure.

**Figure 2:**
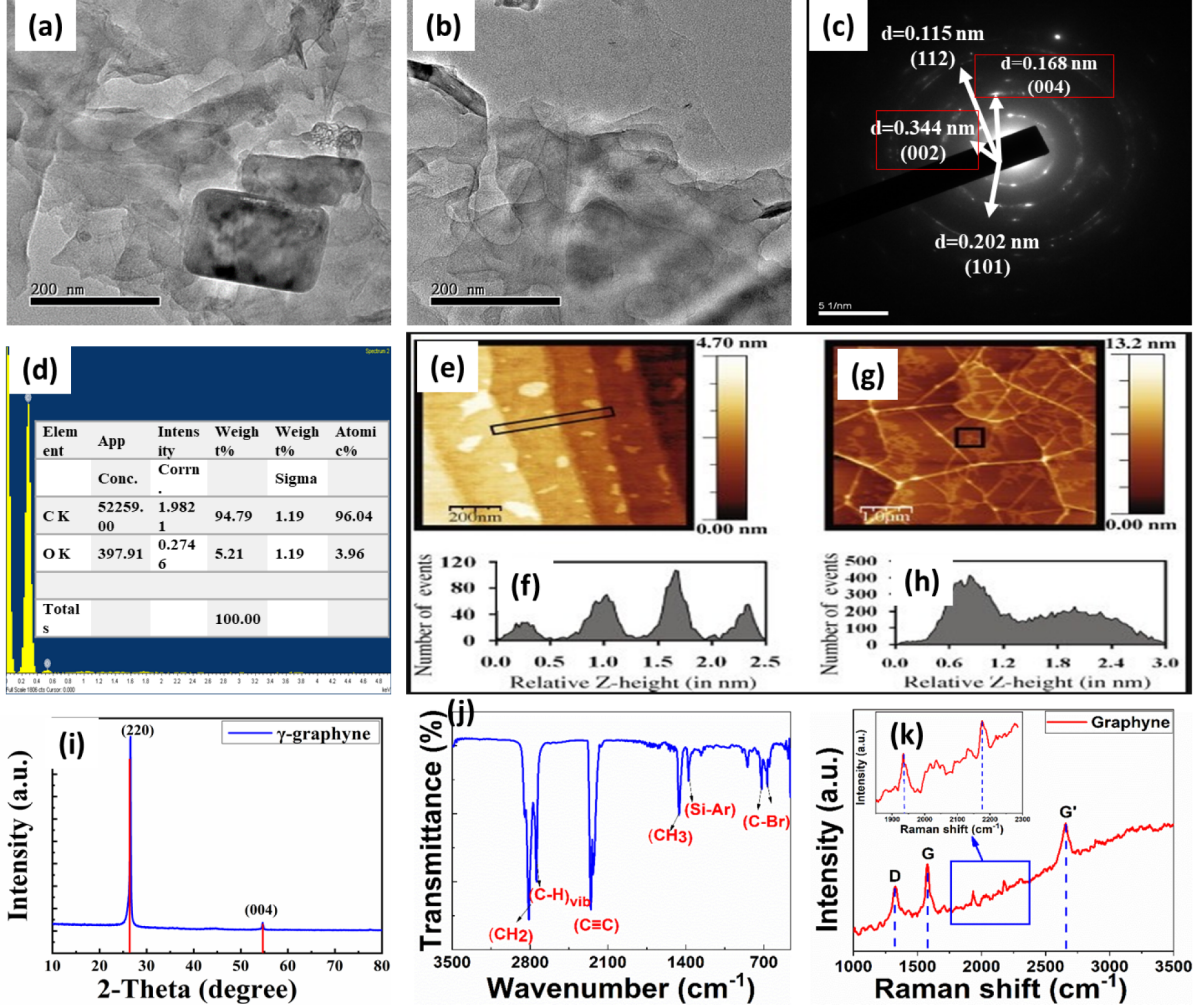
(a,b) HRTEM analysis of graphyne. (c) SAED pattern of graphyne. (d) EDX analysis of graphyne. (e–h) AFM analysis of the graphyne sheet. (i) XRD pattern of graphyne. (j) FTIR analysis. (k) Raman analysis of graphyne.

The lattice fringes of the crystal plane have been analyzed by using the selected area electron diffraction (SAED) pattern, as shown in Figure 2c. The circular pattern of the diffraction spots indicates the polycrystalline nature of the material, where the randomly oriented crystallites diffract the electron beam to produce rings of spots. The bright and distinct spots along the rings further confirm the high degree of crystallinity and the presence of multiple orientations of the crystallites. In particular, the plane (002), having an interplanar spacing of 0.344 nm, represents the 2D planar structure of the graphyne. Furthermore, the plane (004), with a spacing of 0.168 nm, represents the graphitic nature of the graphyne.

Furthermore, the purity of the graphyne has been analyzed using energy-dispersive X-ray spectroscopy (EDX) and is illustrated in Figure 2d. The EDX analysis of the graphyne sample reveals a predominant composition of carbon, accounting for 94.79% by weight and 96.04% by atomic percentage. The remaining composition is oxygen, present in very small amounts. This high carbon content is indicative of the graphyne structure, which is an allotrope of carbon composed of sp and sp^2^-hybridized carbon atoms, forming a two-dimensional network. The minimal oxygen presence could be attributed to surface oxidation or impurities introduced during the synthesis or sample preparation process. The high carbon percentage and the characteristic graphyne structure suggest that the material retains its expected chemical composition, with oxygen being a minor component likely located at defect sites or edges of the graphyne layers. This composition is consistent with the typical characteristics of graphyne, which is designed to maximize carbon content for its unique electronic and structural properties.

Atomic force microscopy (AFM) analysis of the nanoscale surface morphology of graphyne is illustrated in Figure 2f. The provided AFM image elucidates the topographical features of a graphyne sample, with the height variations represented by a color gradient ranging from −2.0 nm (dark regions) to 4.6 nm (light regions). The inset graph showcases a height profile extracted along a marked line in the main image, revealing discrete vertical displacements of approximately 1.5 nm, indicative of atomic layer steps or topographical defects intrinsic to the graphyne structure. These height variations can be attributed to the material’s intrinsic properties, such as its layered configuration or the presence of surface imperfections and adsorbates. The detailed surface mapping afforded by AFM is instrumental in characterizing the nanoscale architecture of graphyne, facilitating a deeper understanding of its structural integrity, defect distribution, and surface phenomena. This information is vital for optimizing the material for advanced applications in nanoelectronics, sensing technologies, and other domains where the precise surface characteristics of graphyne play a critical role in performance and functionality.

The X-ray diffraction (XRD) spectrum of γ-graphyne exhibits distinct diffraction peaks at approximately 28° and 55° in the 2θ range, corresponding to the (220) and (004) crystallographic planes, respectively, as shown in Figure 2i. The pronounced intensity and sharpness of the (220) peak indicate a high degree of crystallinity and a well-ordered lattice structure, characterized by the unique arrangement of sp- and sp^2^-hybridized carbon atoms inherent to γ-graphyne. The less intense (004) peak, while still indicative of a coherent structure, suggests a lower prevalence of this plane within the sample. The FTIR spectrum of graphyne provides crucial insights into the vibrational modes of its molecular structure, highlighting the unique hybridization states of its carbon atoms, as shown in Figure 2j. The prominent peaks in the region of 2100–2250 cm^–1^ are indicative of C≡C stretching vibrations, confirming the existence of acetylenic linkages within the graphyne framework. Additionally, the C=C stretching vibrations, typically observed around 1500–1600 cm^– 1^, further corroborate the presence of sp^2^-hybridized carbon atoms. The intricate pattern of peaks within the fingerprint region (600–1500 cm^–1^) reflects various bending and stretching modes, offering a comprehensive overview of the structural integrity and bonding characteristics of graphyne.

The Raman spectrum depicted is indicative of graphyne, featuring characteristic peaks that provide insights into its structural properties, as shown in Figure 2k. The D band (*I*_D_) at approximately 1350 cm^–1^ signifies the presence of defects and disorder within the sp- and sp^2^-hybridized carbon networks, revealing imperfections or edge effects in the material. The G band (*I*_G_) around 1580 cm^–1^ corresponds to the *E*_2g_ phonon mode at the Brillouin zone center, indicative of the in-plane vibrations of sp^2^-bonded carbon atoms, a hallmark of graphitic materials, confirming the presence of sp^2^ hybridization in the graphyne structure. The calculated *I*_D_/*I*_G_ ratio was found to be 0.91. Additionally, the observed peaks at 1924 cm^–1^ and 2177 cm^–1^ correspond to the Y band, confirming the presence of diyne linkage vibrations. The G’ band (*I*_G’_), located near 2700 cm^–1^, is the second-order harmonic of the D band and is sensitive to the stacking order and number of layers, with its prominence and sharpness suggesting high crystallinity and a well-ordered arrangement. The intensity ratios and peak profiles, such as the *I*_D_/*I*_G_ ratio, offer quantitative measures of the defect density and overall quality of the graphyne, with a higher ratio indicating greater disorder and a prominent G’ band denoting fewer layers and superior crystalline structure.

Nuclear magnetic resonance (NMR) spectroscopy provides detailed insights into the structural and electronic properties of graphyne, as illustrated in S5, which is a carbon allotrope composed of sp- and sp^2^-hybridized carbon atoms. In the ^13^C NMR spectrum, sp^2^-hybridized carbon atoms resonate in the range of 110–150 ppm, indicative of their deshielded environment due to the pi-electron cloud, while sp-hybridized carbons resonate between 60 and 90 ppm, reflecting their linear electronic structure. The relative intensities of these peaks can be integrated to quantify the ratio of sp to sp^2^ carbons, confirming graphyne’s stoichiometry. Spin–spin coupling constants (J-coupling) provide connectivity information, and relaxation times (*T*_1_ and *T*_2_) offer insights into the dynamics and mobility of the carbon atoms.

### Theoretical Model

Figure 3a illustrates the configuration and alignment of graphyne sheets for the gas sensor. In this model, two different graphyne sheets *S*_1_ and *S*_2_ having different lengths (*l*_1_ and *l*_2_) and widths (*W*_1_ and *W*_2_), respectively, have been attached to each other randomly, as illustrated in Figure 3b. Due to their differing lengths, the donor and acceptor levels along the surface are different, creating a potential along the junctions. Considering the donor and acceptor levels as *N*_A1_ and *N*_D1_ for sheet S_1_ and *N*_A2_ and *N*_D2_ for sheet S_2_, to understand the junction potential across the junction, we start with Poisson’s equation:

4 $$\frac{dE}{dx}=\frac{\rho }{\kappa \varepsilon_{0}}$$

5 $$\rho =q[p\left(x\right)-n\left(x\right)+N_{D1}^{+}-N_{A1}^{-}]$$

**Figure 3:**
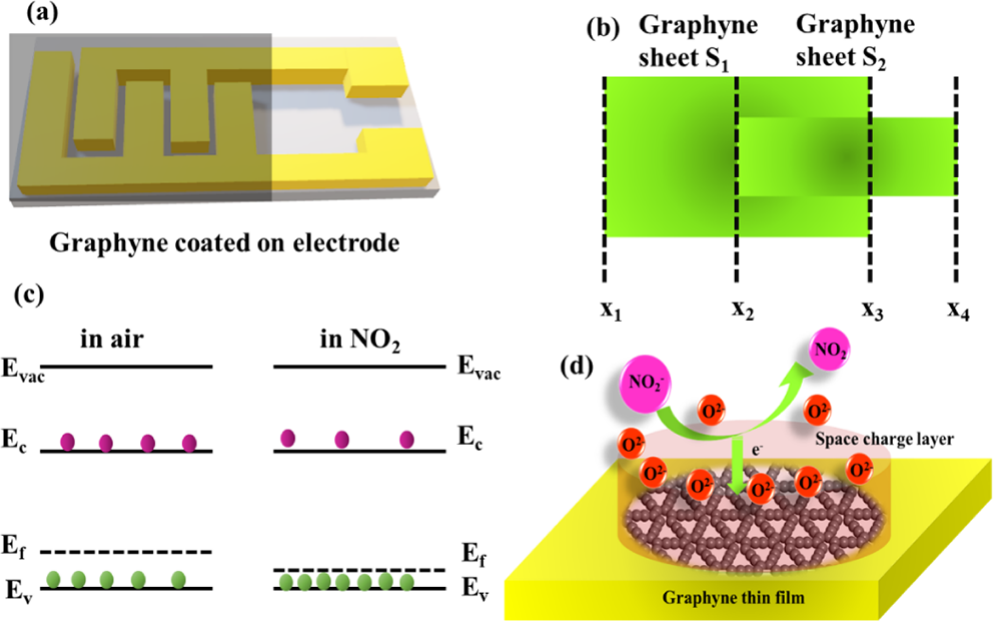
(a) Fabrication of a device using graphyne. (b) Alignment of sheets in the device. (c) Variation of the band gap during interaction. (d) Change in electron density during adsorption.

The graphyne sheets have p-type characteristics, so we are neglecting the minority charge, i.e., $N_{D1}^{+}$ and only considering the majority carrier $N_{A1}^{-}$ . Furthermore, we are analyzing the charge density along the sheet:

6 $$\rho \left(x\right)=0\;x_{1}<x<x_{2}$$

7 $$\rho \left(x\right)=\left(-qN_{A1}\right)+\left(-qN_{A2}\right){\;x}_{2}<x<x_{3}$$

8 $$\rho \left(x\right)=0{\;x}_{3}<x<x_{4}$$

By introducing these values into Poisson’s equation, it becomes

9 $$\frac{dE}{dx}=\frac{-\left(q_{1}+q_{2}\right)\left(N_{A1}+N_{A2}\right)}{\kappa_{\rho }\varepsilon_{0}}{\;x}_{2}<x<x_{3}$$

By integrating both sides, we get

10 $$\int_{0}^{V\left(x\right)}dV\left(x\right)=\frac{-\left(q_{1}+q_{2}\right)\left(N_{A1}+N_{A2}\right)}{\kappa_{\rho }\varepsilon_{0}}\int_{x_{2}}^{x_{3}}\left(x_{3}-x_{2}\right)dx$$

11 $$V\left(x\right)=\frac{-\left(q_{1}+q_{2}\right)\left(N_{A1}+N_{A2}\right)}{2\kappa_{\rho }\varepsilon_{0}}{\left(x_{3}-x_{2}\right)}^{2}$$

Now evaluate the condition where no dipole exists at boundary

12 $$V\left(O^{-}\right)=V\left(O^{+}\right)$$

This is equivalent potential across the graphyne layer during the formation of the multilayer model. Now, considering the interaction of the gas molecule NO_2_ (guest molecule), due to the high oxidation nature of NO_2_, it easily gets oxidized by taking electrons from the atmosphere and is converted into NO_2_^–^. Therefore, the equivalent potential on the NO_2_ ion was considered as $-q_{h}^{*}$ . The overall change in the potential may be considered as

13 $$V^{\prime }\left(x\right)=\frac{-\left(q_{1}+q_{2}-q_{h}\right)\left(N_{A1}+N_{A2}-N_{AH}\right)}{2\kappa_{\rho }\varepsilon_{0}\varepsilon_{g}}{\left(x_{3}-x_{2}\right)}^{2}$$

Furthermore, the sensor response of the sensor could be written as the ratio of the change in the potential gradient in the presence of NO_2_ to the referenced potential gradient.

14 $$SR\left(Sensor\;response\right)=\frac{\frac{d^{2}V’\left(x\right)}{dx^{2}}-\frac{d^{2}V\left(x\right)}{dx^{2}}}{\frac{d^{2}V\left(x\right)}{dx^{2}}}$$

15 $$SR=\left\{\frac{-q}{\kappa_{p}\varepsilon_{0}}\left[n_{i}e^{-q\left(v-v_{p}-\phi_{p}-\phi_{NO2}\right)/K_{B}T}+DOP\left(x\right)\right]-\frac{-q}{\kappa_{p}\varepsilon_{0}}\left[n_{i}e^{-q\left(v-\phi_{p}\right)/K_{B}T}+DOP\left(x\right)\right]\right\}/\left\{\frac{-q}{\kappa_{p}\varepsilon_{0}}\left[n_{i}e^{-q\left(v-\phi_{p}\right)/K_{B}T}+DOP\left(x\right)\right]\right\}$$

On solving this, we get

16 $$SR=\frac{e^{q\phi_{NO2}}}{\frac{\kappa_{p}\varepsilon_{0}}{q}\frac{d^{2}V\left(x\right)}{dx^{2}}}$$

From the above equation, it has been observed that the response is directly dependent on the potential create by the NO_2_ ions, which also affect the band levels of graphyne, as shown in Figure 3c. Therefore, when the number of ions is increased, the sensor response of the sensor should increase, as shown in Figure 3d.

### Gas Sensing Results

Figure 4a–d illustrates the dynamic response of γ-graphyne sensors to different concentrations of NO_2_ gas under controlled relative humidity (RH) conditions. In Figure 4a, the sensor’s resistance response to 25 ppb NO_2_ gas is shown. The sensor’s resistance decreases as NO_2_ gas is introduced (gas on) and reaches an equilibrium state, where the adsorption rate equals the desorption rate. When the gas is turned off, the desorption process begins, and the resistance returns to its initial position, indicating a reversible interaction with the gas. Figure 4b demonstrates the sensor’s response to 50 ppb of NO_2_ gas. Similar to (4a), the resistance shows a reversible decrease upon NO_2_ exposure. Figure 4c,d displays the sensor’s response to 75 and 100 ppb NO_2_ gas, respectively. The resistance continues to exhibit a reversible trend but with a more pronounced change due to the higher NO_2_ concentration. In all cases, the blue line represents the relative humidity, which remains relatively constant, ensuring that the observed resistance changes are primarily due to the NO_2_ gas and not variations in humidity. It is noted that the observed baseline fluctuations are primarily due to the presence of defect sites on the surface of graphyne. NO_2_ molecules can easily adsorb onto these defect sites but are more difficult to desorb due to the strongly bonded chemisorption process. This strong chemisorption contributes to the minor baseline drift observed in the measurements.

**Figure 4:**
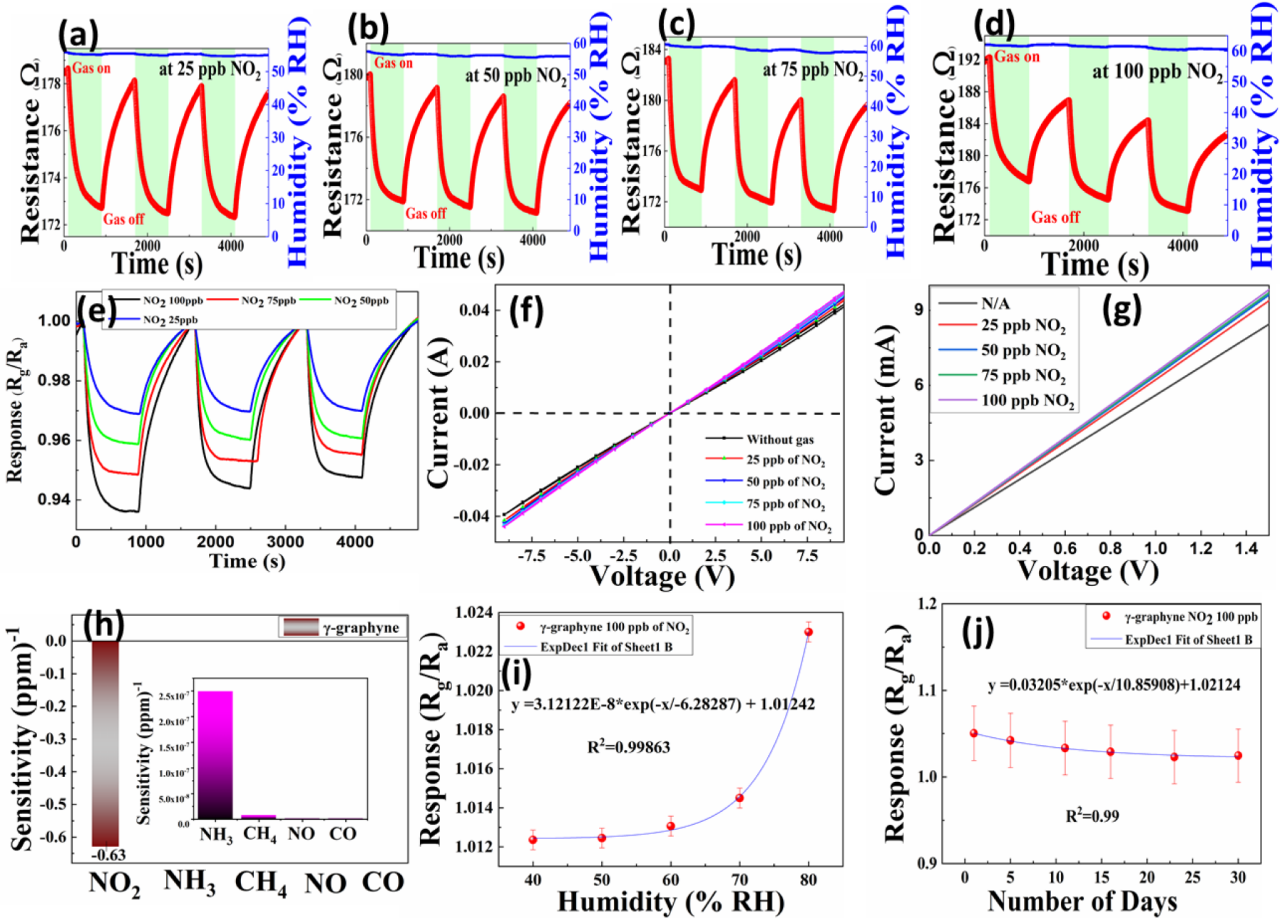
Variation in the resistance of graphyne-based thin film at (a) 25 ppb, (b) 50 ppb, (c) 75 ppb, (d) 100 ppb. (e) Comparison of the variation in sensing response. (f) IV characteristics of graphyne at different concentrations. (g) Current density vs voltage at different concentrations. (h) Selectivity of graphyne toward different gases. (i) Sensor response at different levels of humidity. (j) Long-term stability test of graphyne.

Figure 4e presents the normalized response (*R*_g_/*R*_a_) of the γ-graphyne sensor as a function of time under varying concentrations of NO_2_ (25, 50, 75, and 100 ppb). The response curves indicate that the sensor’s sensitivity increases with NO_2_ concentration. The higher the NO_2_ concentration, the more significant the drop in *R*_g_/*R*_a_, showing that the sensor is more responsive at higher gas concentrations. The variations in the slope of the IV diagram of graphyne at different concentrations are shown in Figure 4f. As the concentration of NO_2_ increases (red, blue, cyan, and magenta for 25, 50, 75, and 100 ppb, respectively), there is a slight increase in the slope of the IV curve, implying a marginal increase in current for a given voltage. Since NO_2_ is a highly oxidizing gas, when it comes in contact with air, it takes some electrons from the air and becomes charged. Furthermore, it interacts with the adsorbed oxygen species on the surface of graphyne. This interaction leads to the release of some free electrons to graphyne, converting NO into an oxygen molecule. The variation in the output current in the presence of various NO_2_ levels below a 1.5 V operating voltage has been depicted in Figure 4g.

Furthermore, to examine the selectivity of the graphyne-based sensor, the thin film was placed under the atmosphere of different gases, as depicted in Figure 4h. The highest sensitivity (−0.63 ppm^–1^) was found for NO_2_ gas. However, the sensitivity for other gases like NH_3_ and CH_4_ was 2.4 × 10^–6^ ppm^–1^ and 7.38 × 10^–9^ ppm^–1^, respectively. Figure 4i shows the relationship between the sensor’s response and relative humidity. The response is plotted as a function of RH (%), showing an exponential increase in the response with increasing humidity. This suggests that the sensor’s performance is highly sensitive to humidity changes at higher RH levels, with a fitting curve demonstrating the exponential trend. These figures collectively highlight the γ-graphyne sensor’s effectiveness in detecting low concentrations of NO_2_ gas and its dependence on relative humidity. The reversible resistance changes and the sensor’s heightened response at higher NO_2_ concentrations and humidity levels underscore its potential for environmental monitoring applications. The long-term stability test is illustrated in Figure 4j. A comparison of the gas sensing response with another reported sensor is provided in Table 1.

**Table 1:** Comparative Study of Graphyne Sensors with Previously Published Work

S. No.	Materials	Type of gas	concentration	Operating temperature (°C)	Sensor Response	Response/Recovery time (s)	ref.
1	IGZO-ZnO	NO_2_	5 ppm	250	48	172/295	^33^
2	rGO-SnS_2_	NO_2_	5 ppm	150	32	50/48	^34^
3	MoS_2_/rGO	NO_2_	3 ppm	160	1.24	8/20	^35^
4	MoS_2_/ZnS	NO_2_	5 ppm	RT	7.2	246/312	^36^
5	WS_2_/GA	NO_2_	2 ppm	180	2	NA	^37^
6	SnO_2_-rGO	NO_2_	0.5 ppm	50	1.5	135/200	^38^
7	MoS_2_/PbS	NO_2_	10 ppm	RT	6.15	15/62	^39^
8	SnO_2_/MoS_2_	NO_2_	10 ppm	RT	0.28	400/180	^40^
9	SnS_2_@SnO_2_	NO_2_	0.2 ppm	RT	5.3	950/1160	^41^
**10**	**Graphyne**	**NO**_**2**_	**25 ppb**	**RT**	**1.05**	**57/185**	**This work**

The comparative analysis presented in Table 1 clearly demonstrates the advantages of the proposed graphyne-based NO_2_ sensor over previously reported materials. Most conventional NO_2_ sensors, such as IGZO–ZnO, rGO–SnS_2_, and MoS_2_/rGO, require elevated operating temperatures ranging from 150 to 250 °C to achieve acceptable sensitivity, which significantly increases power consumption and limits their applicability in portable and wearable devices. Even some heterostructures that operate at room temperature, such as SnO_2_/MoS_2_ and SnS_2_@SnO_2_, suffer from either extremely low sensor response (0.28 at 10 ppm) or very long response/recovery times (950/1160 s), making them unsuitable for rapid real-time monitoring. Furthermore, the detection limits of most existing sensors remain at parts-per-million levels, which is inadequate for applications requiring ultrasensitive detection of NO_2_ in environmental or biomedical contexts. In contrast, the graphyne-based sensor reported in this work achieves an exceptionally low detection limit of 25 ppb at room temperature without the need for external heating, offering both energy efficiency and operational simplicity. Additionally, the proposed sensor exhibits a balanced performance, with a high response (1.05) and reasonable response/recovery times (57/185 s), outperforming most room-temperature sensors while avoiding the sensitivity-speed trade-off common in previous studies. This superior performance can be attributed to the unique electronic structure of graphene, which provides abundant active sites and enhanced charge transfer interactions with NO_2_ molecules, making it a promising candidate for next-generation low-power, highly sensitive gas sensors.

The gas sensing curve confirms the p-type adsorption behavior of graphyne. The observed gas sensing characteristics of graphyne can be attributed to the junctions formed across the grain boundaries between different layers. These grain boundaries play a crucial role in charge accumulation, which significantly influences the adsorption of gas molecules. In this experiment, the graphyne thin film was placed inside a gas sensing chamber, where it initially interacted with atmospheric oxygen, leading to the formation of oxygen species on its surface, as given as

17 $$O_{2}+e^{-}\to O_{2}^{-}$$

18 $$O_{2}^{-}\to 2O^{-}$$

19 $$Graphyne+O^{-}\to Graphyne\cdot \cdot \cdot O^{-}$$

Upon the introduction of NO_2_ into the chamber, this highly oxidizing gas interacted with the existing atmospheric gases, extracting electrons due to its strong electron affinity and becoming charged in the process. Subsequently, the charged NO_2_ species interacted with the preadsorbed oxygen species on the graphyne surface. This interaction resulted in the reduction of NO_2_ to NO, and the concurrent formation of the O_2_ molecules, which released free electrons into the graphyne sheets.

20 $$NO_{2}+e^{-}\to NO_{2}^{-}$$

21 $$Graphyne\cdot \cdot \cdot O^{-}+NO_{2}^{-}\to Graphyne+NO+O_{2}+3e^{-}\left(graphyne\right)$$

The injection of these active electrons into the graphyne network facilitated an increase in the material’s conductivity, manifesting as a decrease in resistance during the sensing measurements. This mechanism highlights the pivotal role of charge transfer processes at the grain boundaries in detecting the gas sensing response of graphyne.

Furthermore, the interaction of graphyne with the NO_2_ molecule has been analyzed by using the Material Studio software package. First, the graphyne structure was optimized in a 2 × 2 supercell for calculation convenience, as illustrated in Figure 5. The graphyne contains 12 carbon atoms in each unit cell, with a sp and sp^2^ hybridization ratio of 1:1. Due to the deformative structure of graphyne, the bond length of the hexagon R1 was considered as 1.425 *AÅ*. In the acrylic link, the C_sp_-C_sp_ and C_sp_-C_sp2_ bond lengths were 1.226 *AÅ* and 1.407 *A*Å, respectively. The results observed in this structure are in good agreement with previously published articles. The adsorption energy of graphyne interacting with NO_2_ has been calculated by using the formula provided in the equation:

22 $$E_{\left(ads\right)}=E_{Graphyne+NO_{2}}-E_{Graphyne}-E_{NO_{2}}$$

**Figure 5:**
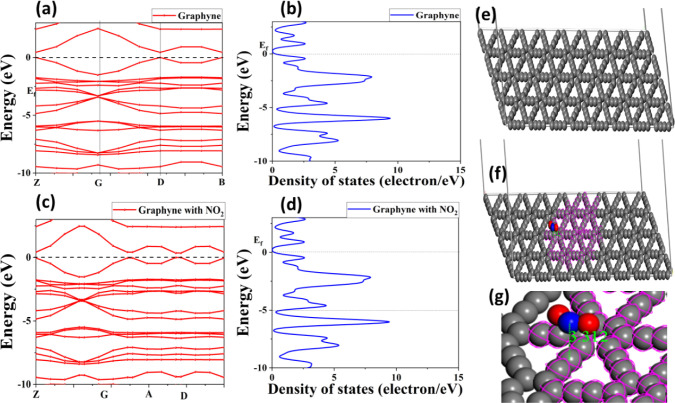
(a) Band structure of graphyne. (b) DOS of pristine graphyne. (c) Band structure of graphyne with NO_2_. (d) DOS of graphyne with NO_2_ (e) Optimized structure of graphyne. (f) Graphyne with NO_2_. (g) Interatomic distance between NO_2_ and the graphyne sheet.

In order to investigate the behavior of NO_2_ on the surface of graphyne, different sites for adsorption have been chosen, and the structure has been optimized in each configuration. The adsorption energy of graphyne toward NO_2_ has been found to be −1.3 eV. During the adsorption process, a small change in charge has been observed. These variations in charge are responsible for the change in band level and the decrease in the band gap of the material.

Figure 5 depicts the band structure and density of states (DOS) of graphyne, both in its pristine form and when adsorbed with NO_2_ molecules. Figure 5a shows the band structure of pristine graphyne. The horizontal axis represents different high-symmetry points in the Brillouin zone (Z, G, D, and B), while the vertical axis represents the energy levels in electron volts (eV). The dashed line at 0 eV corresponds to the Fermi level (*E*_f_). In this graph, it has been observed that the valence bands touch the Fermi level, suggesting that pristine graphyne has a p-type semiconducting nature with a band gap of about 0.46 eV. Furthermore, Figure 5b illustrates the DOS of pristine graphyne. The vertical axis represents the energy levels, and the horizontal axis represents the density of states (electrons per eV). The DOS near the Fermi level often shows a significant number of states, which indicates that graphyne may exhibit p-type semiconducting behavior. This means that electrons can easily be excited into conduction bands, allowing for electrical conductivity.

Figure 5c shows the band structure of graphyne when NO_2_ molecules are adsorbed onto its surface. The general shape of the band structure has been altered compared to the pristine case. The interaction with NO_2_ has introduced new states or shifted the existing ones, possibly opening up a band gap or modifying the electronic properties near the Fermi level. This change indicates that NO_2_ adsorption significantly affects the electronic structure of graphyne. Figure 5d illustrates the DOS of graphyne after NO_2_ adsorption. Compared to the DOS of pristine graphyne, the DOS plot here shows changes in the distribution of states around the Fermi level. There may be a reduction in the DOS at the Fermi level, indicating a reduction of band gap, which increases the conductivity due to NO_2_ adsorption. The changes in the DOS reflect the impact of NO_2_ on the electronic structure, potentially making the material more semiconducting.

Figure 5d–f displays structural models of graphyne, both in its pristine form (top) and with NO_2_ adsorbed (middle and bottom). The NO_2_ molecule, shown in the middle image, likely interacts with the carbon atoms of graphyne, possibly through charge transfer or chemical bonding. The bottom image provides a closer view of the adsorption site, highlighting the interaction between the NO_2_ molecule and the graphyne lattice.

### Gas Sensing Analysis Using Machine Learning

Following the gas sensing curves for the different gas analytes by using graphyne, the response of each analyte gas at different concentrations was measured over 20 consecutive cycles. In each individual measurement, there are two distinct phases: the gas exposure phase and the flushing phase.^42^ As shown in Figure 4h, the response toward NO_2_ is significantly higher in comparison to the other gases. Furthermore, the measurement of each concentration of NO_2_ gas, ranging from 25 to 100 ppb, was characterized by the developed gas sensor. The schematic of the gas sensing response used to measure different functions is shown in Figure S6. Machine learning is essential for extracting insights, predicting outcomes, and enabling intelligent decision-making from complex and large-scale data.^43,44^ All the data were analyzed by using unsupervised machine learning (principal component analysis, PCA) and supervised machine learning (linear discriminant analysis, LDA).^45^

Figure 6a illustrates the linear regression analysis comparing the predicted and actual gas sensing responses across various NO_2_ concentrations. The close alignment of data points along the regression line indicates a strong correlation, demonstrating that the predicted response closely matches the experimentally measured values. This high degree of agreement validates the reliability and accuracy of the sensor, confirming its suitability for NO_2_ detection across different concentration levels. Similarly, Figure 6b presents the variation between the true and predicted responses, showcasing the consistency of the predictive model. The minimal deviation observed further reinforces the sensor’s capability to provide accurate real-time gas concentration estimations, making it a robust candidate for practical NO_2_ sensing applications. Figure 6c presents principal component analysis (PCA) results for NO_2_ gas sensing at different concentrations, illustrating the sensor’s ability to discriminate between varying levels of NO_2_ exposure.^46^ Figure 6c shows the PCA score plot displaying the clustering of NO_2_ concentrations (15, 25, 50, and 100 ppb) based on their principal components (PC1:65.62%, PC2:32.09%). Each concentration forms a distinct cluster, indicating effective differentiation. The spatial separation among the clusters suggests that the sensing response varies significantly with NO_2_ concentration, demonstrating the sensor’s high selectivity.

**Figure 6:**
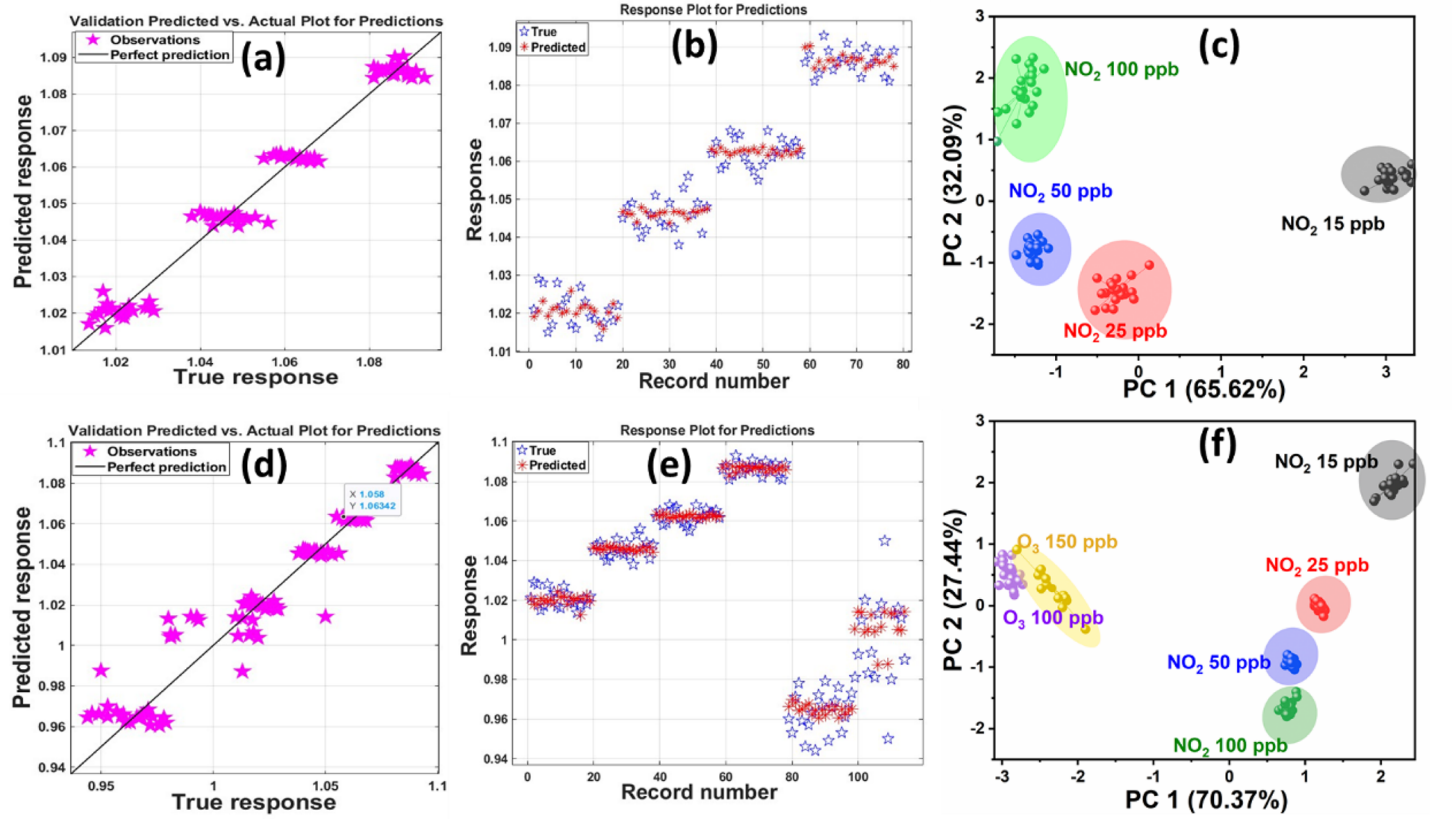
(a) Linear discrimination plot of NO_2_ vs concentrations. (b) True vs actual value prediction using linear regression. (c) Principal component analysis of NO_2_ at different concentrations. (d) Linear discrimination of selectivity of NO_2_. (e) Predicted vs true value at various NO_2_ concentrations. (f) Potential for precise gas detection applications. PCA of the selectivity toward other oxidizing gases.

Figure 6d,e illustrates the selective analysis of gas sensing performance for NO_2_ and O_3_ through linear regression and response variation, respectively. The regression plot in Figure 6d demonstrates a strong correlation between the predicted and actual sensor responses, indicating the reliability and precision of the predictive model in differentiating between the concentrations of NO_2_ and O_3_ at various levels. The clustering of data points along the regression line further validates the sensor’s high accuracy in detecting and quantifying these gases. Figure 6e presents the comparative sensor response variation for NO_2_ and O_3_ gases, showcasing the stability and consistency of the sensor’s output. The close alignment between true and predicted responses suggests that the sensor effectively discriminates between NO_2_ and O_3_, with minimal deviation, reinforcing its suitability for selective gas detection. These results highlight the sensor’s robust performance in accurately monitoring multiple gas species in real-world environmental applications. Figure 6f shows the PCA score plot, which incorporates a higher NO_2_ concentration (150 ppb) with an adjusted variance distribution (PC1:70.37%, PC2:27.44%). The clusters remain well-separated, confirming the sensor’s capability to distinguish NO_2_ levels across a broader concentration range. Notably, the addition of 150 ppb of NO_2_ results in a shifted cluster, suggesting an increasing trend in response with higher gas exposure. These PCA plots effectively validate the sensor’s high selectivity, reproducibility, and capability to resolve NO_2_ concentrations with minimal overlap, confirming its potential for precise gas detection applications.

Figure 7 provides a detailed analysis of the validation and classification performance of a gas sensor by using machine learning models. It evaluates the sensor’s ability to distinguish different NO_2_ concentrations and its cross-selectivity against other interfering gases, ensuring high accuracy and reliability in gas sensing applications.

**Figure 7:**
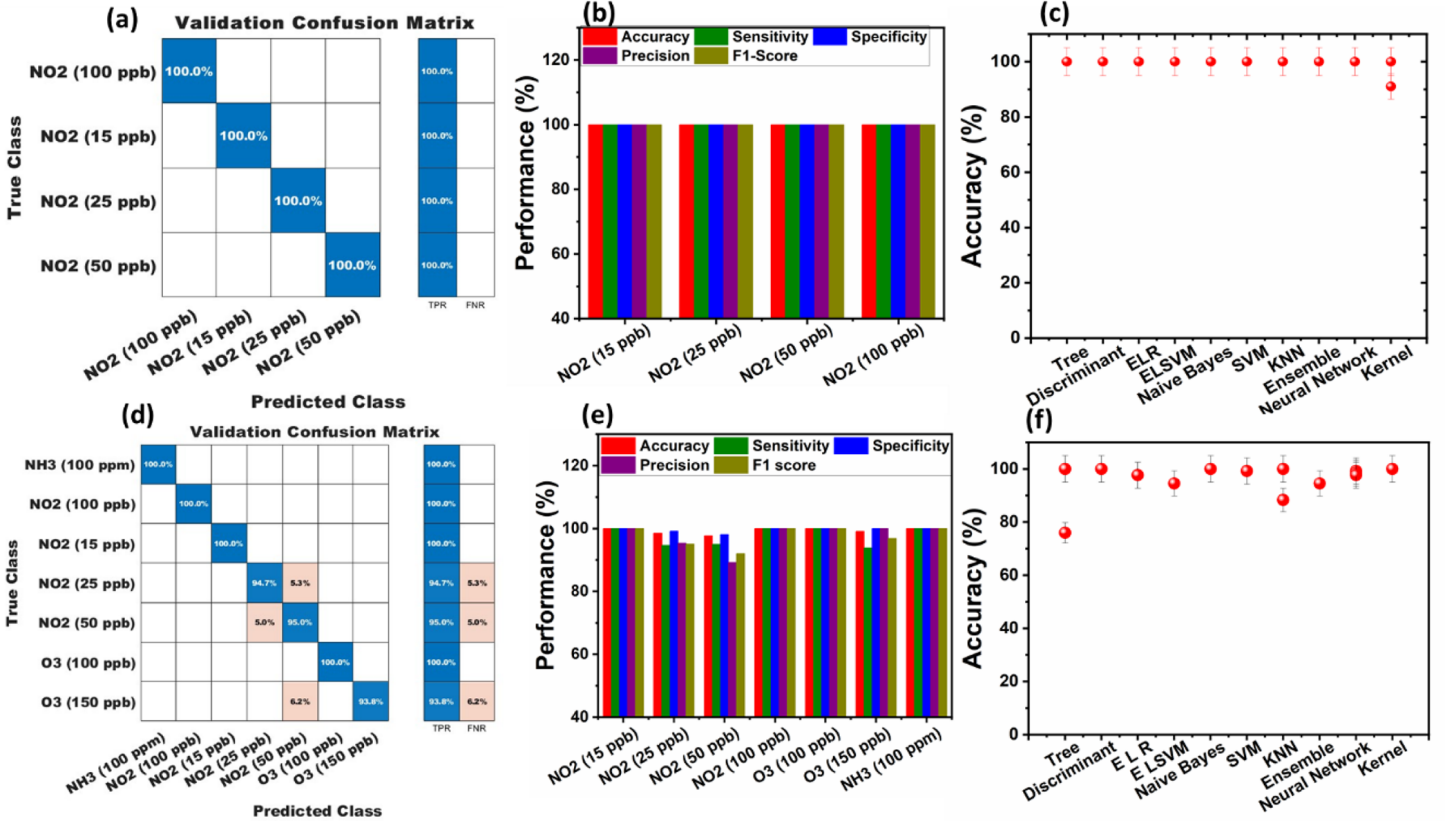
(a) Confusion matrix of NO_2_ at various concentrations using the LDA classifier algorithm. (b) Sensor performance toward NO_2_ by using the hold out cross-validation method. (c) Accuracy results of gas identification with respect to classifier algorithms via the k fold cross-validation method. (d) Confusion matrix of selectivity toward other gases using the LDA classifier algorithm. (e) Sensor performance toward selective oxidizing sensing by using the hold out cross-validation method. (f) Accuracy results of different gas identification with respect to classifier algorithms via the k fold cross-validation method.

The confusion matrices in Figure 7a,d illustrate the classification performance for NO_2_ and multigas environments. In Figure 7a, the sensor demonstrates 100% classification accuracy for NO_2_ at varying concentrations (15, 25, 50, and 100 ppb), with no misclassification observed. This indicates a highly selective and repeatable sensing mechanism. In contrast, Figure 7d extends the classification to NH_3_ (100 ppm) and O_3_ (100 and 150 ppb), where minor misclassifications are observed. NO_2_ at 25 and 50 ppb shows false classification rates of 5.3% and 5.0%, respectively, while O_3_ at 150 ppb exhibits a false classification rate of 6.2%, indicating some overlap in sensor response at higher O_3_ concentrations. However, NH_3_ and high NO_2_ concentrations remain well-differentiated, confirming strong selectivity.

The parameters such as accuracy, precision, sensitivity, specificity, and F1-score for NO_2_ and multigas sensing have been calculated by using the values of true positive (TP), true negative (TN), false positive (FP), and false negative (FN). The corresponding values have been determined using the confusion matrix. These values have been calculated by using eqs 23–27.

23 $$Accuracy=\frac{\left(TP+TN\right)}{\left(TP+FP+TN+FN\right)}$$

24 $$Sensitivity=\frac{TP}{\left(TP+FN\right)}$$

25 $$Specificity=\frac{TN}{\left(TN+FP\right)}$$

26 $$Precision=\frac{TP}{\left(TP+FP\right)}$$

27 $$F1Score=2\times \frac{\left(precision\times sensitivity\right)}{\left(precision+sensitivity\right)}=\frac{2TP}{2TP+FP+FN}$$

The performance metrics in Figure 7b,e further quantify the classification accuracy, precision, sensitivity, specificity, and F1-score for NO_2_ and multigas sensing. In Figure 7b, all performance indicators remain consistently high (∼100%), reflecting exceptional sensor stability, repeatability, and minimal variation across multiple measurements. For multigas classification in Figure 7e, a slight decrease in specificity and sensitivity is observed for certain NO_2_ concentrations, particularly 25 and 50 ppb, which aligns with the minor misclassification noted in Figure 7d. Despite this, overall performance remains above 90%, demonstrating the sensor’s strong gas discrimination capability.

The machine learning model comparison in Figure 7c,f evaluates different classification algorithms, including tree discriminant, elastic logistic regression (ELR), elastic support vector machine (ELSVM), naïve Bayes, SVM, K-nearest neighbors (KNNs), ensemble, neural network, and kernel-based methods. In Figure 6c, all models achieve near-100% accuracy for NO_2_ classification, confirming the effectiveness of the data set and feature extraction process. For multigas classification in Figure 6f, slight performance variations are noted, with KNN, ensemble, and neural network classifiers achieving the highest accuracy, while tree discriminant and naïve Bayes exhibit slightly lower precision due to their reliance on simpler decision boundaries.

In conclusion, the sensor demonstrates high selectivity, stability, and classification accuracy for the measurement of NO_2_ and other gases. Despite minor misclassifications at lower NO_2_ concentrations and higher O_3_ levels, the overall performance remains robust across multiple machine learning frameworks. The findings validate the sensor’s potential for real-time gas monitoring applications, ensuring reliable environmental and industrial gas detection.

## Conclusions

In conclusion, graphyne was successfully synthesized by using a facile chemical exfoliation method. Characterization confirmed the formation of layered graphyne with varying sheet lengths. The reaction mechanism was demonstrated by using DFT calculations. Theoretical calculations revealed that these variations in sheet length generate different potentials, with junctions playing a critical role in gas molecule adsorption. Experimental results demonstrated strong NO_2_ adsorption, with a sensor response of 1.05 at 25 ppb and a limit of detection of 0.45 ppb. The sensor exhibited fast response (53 s) and recovery times (185 s). The selectivity of the sensor is very high, and it also has long-term stability. DFT calculations provided deeper insight into gas adsorption properties, supporting the experimental findings. We demonstrated a highly selective and sensitive gas sensor capable of distinguishing varying concentrations of NO_2_ while maintaining strong selectivity against interfering gases such as NH_3_ and O_3_. The sensor exhibited 100% classification accuracy for NO_2_ concentrations ranging from 15 to 100 ppb, as confirmed by the confusion matrix analysis. In the presence of multiple gases, minor misclassifications were observed, particularly at NO_2_ 25 ppb (5.3%) and NO_2_ 50 ppb (5.0%), while O_3_ at 150 ppb exhibited a 6.2% misclassification rate, indicating some spectral overlap in the sensor’s response. However, NH_3_ remained distinctly classified, ensuring strong selectivity. Overall, our findings highlight the sensor’s high selectivity, stability, and classification accuracy, demonstrating its potential for real-time gas monitoring in environmental and industrial applications. Future work will focus on further optimizing the sensor’s response characteristics to enhance discrimination against structurally similar interfering gases, ensuring even greater reliability for practical deployment.

## Research Highlights

Achieved the successful synthesis of γ-graphyne via a facile chemical exfoliation approach.
Addressed the challenges of graphene tuning through structural modifications.
Developed an ultrasensitive NO_2_ sensor utilizing graphyne nanosheets (NSs).
Elucidated the gas sensing mechanism through theoretical modeling.
Machine learning algorithms were trained to recognize and identify different gases.
Quantum modeling, charge transport physics, and machine learning synergize to enable ultrasensitivity and selectivity in next-generation gas sensors.

## Supplementary Material



## Notes

Supporting Information includes the synthesis procedure, energy band diagrams, and a detailed description of the gas sensing setup; it also provides HRTEM and NMR images, along with the energy level variations upon NO_2_ adsorption; additionally, it presents the parameters employed for machine learning model development (PDF)
