# Supplementary material for: γ‑Graphyne as a Functional 2D Nanoarchitectonics for Room-Temperature Chemiresistive–Potentiometric Sensing Interfaces
Source: ACS Sens. 2025 Oct 2;10(10):7508–20. doi: 10.1021/acssensors.5c01507 (PMC12560122; doi:10.1021/acssensors.5c01507)
Supplement: Supplementary file 1 [file se5c01507_si_001.pdf]

Supporting Information

ϒGraphyne as a Functional 2D Nano architectonics for Room-Temperature Chemiresistive–Potentiometric Sensing Interfaces

Utkarsh Kumar,[a,b] Pei-Ying Wu,[c] Chun-En Lin,[a] Zu-Yin Deng,[a] Ren-Jang Wu,[d] Kuen-Lin Chen,[a] Wen-Min Huang,[a] and Chiu-Hsien Wu[a,c,*]

[a]Department of Physics, National Chung Hsing University, Taichung 402, Taiwan

[b]i - Center for Advanced Science and Technology (i CAST), National Chung Hsing University, Taichung 402, Taiwan

[c]Institute of Nanoscience, National Chung Hsing University, Taichung 402, Taiwan

[d]Department of Applied Chemistry, Providence University, Taichung 43301, Taiwan

Corresponding author: Chiu-Hsien Wu; email: chwu@phys.nchu.edu.tw

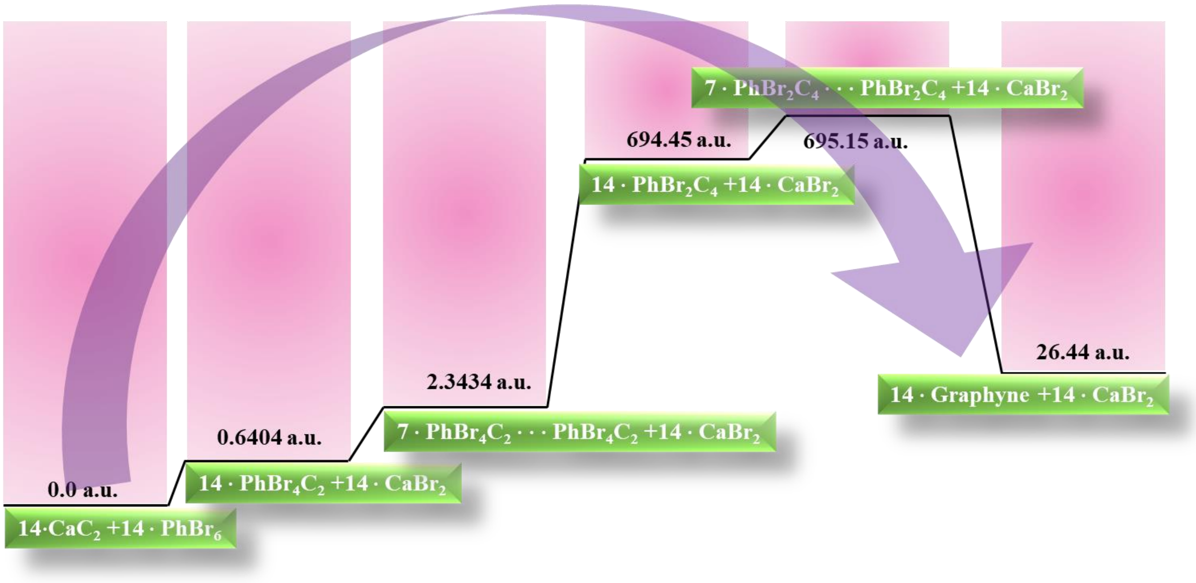

Fig. S1 variation in the energy in each steps of synthesis.

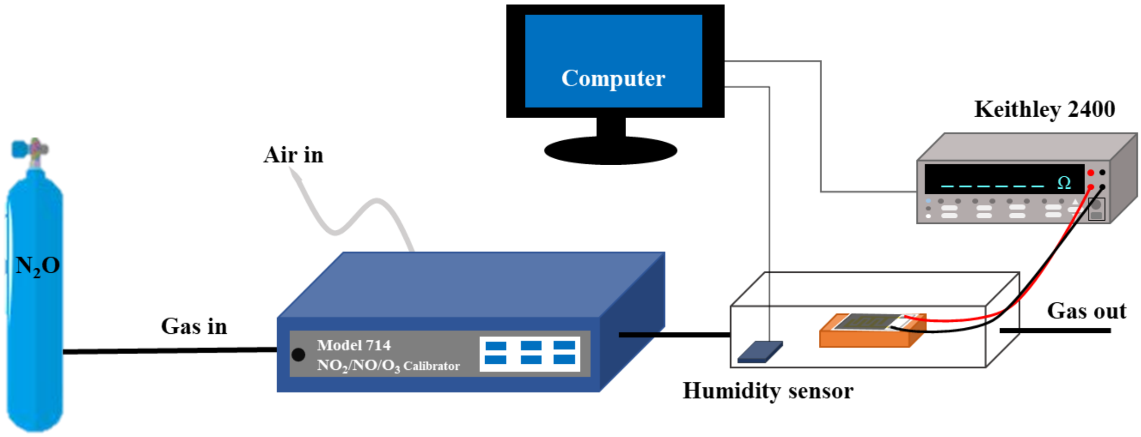

Fig. 2 Schematic diagram of gas sensing diagram

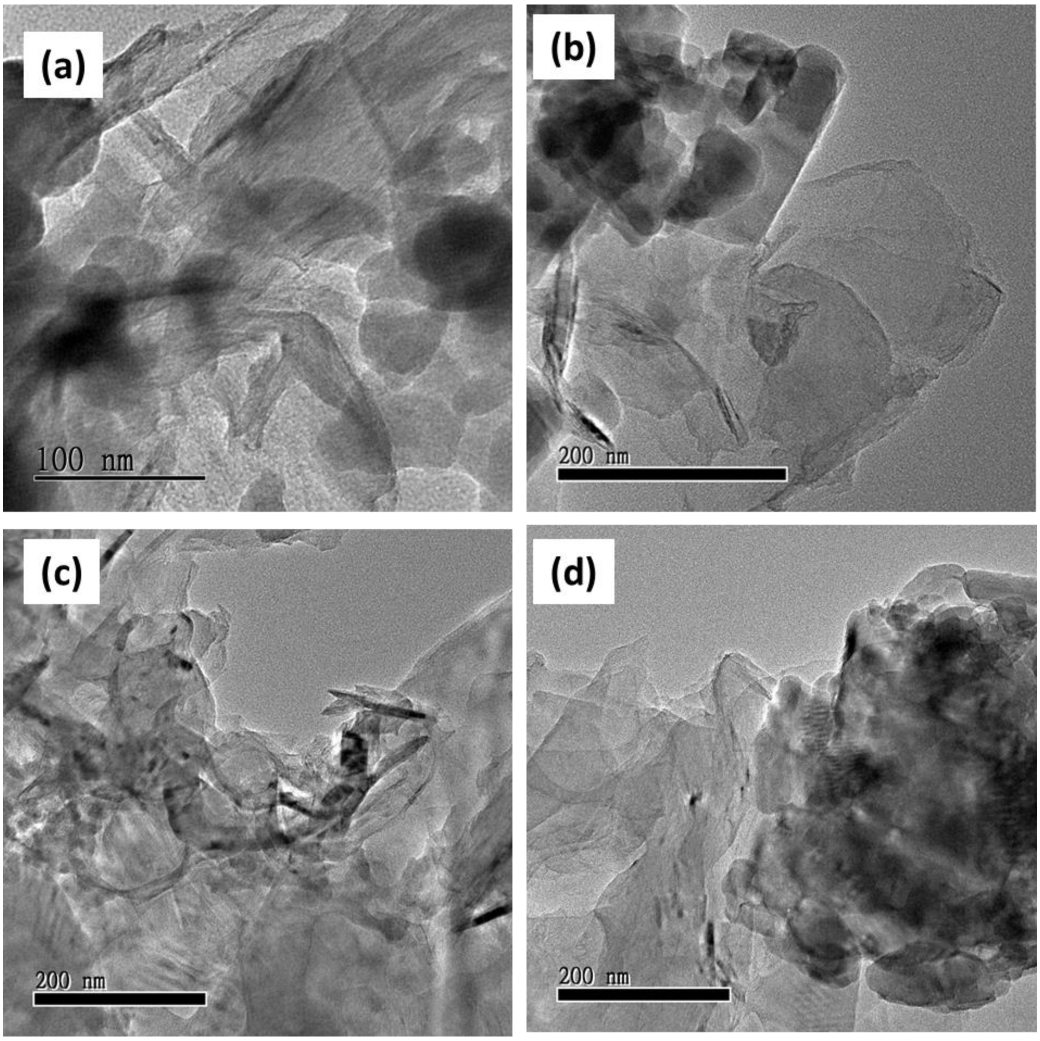

Fig. S3 HRTEM analysis of graphyne at different scale

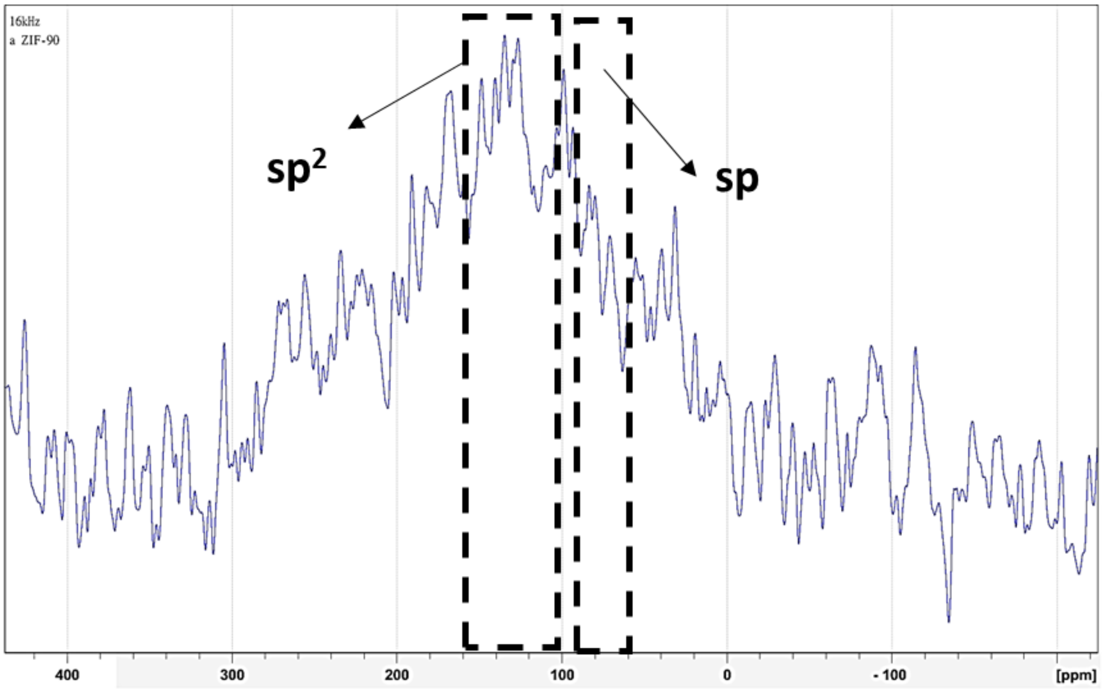

Fig. S4 NMR spectra of Graphyne

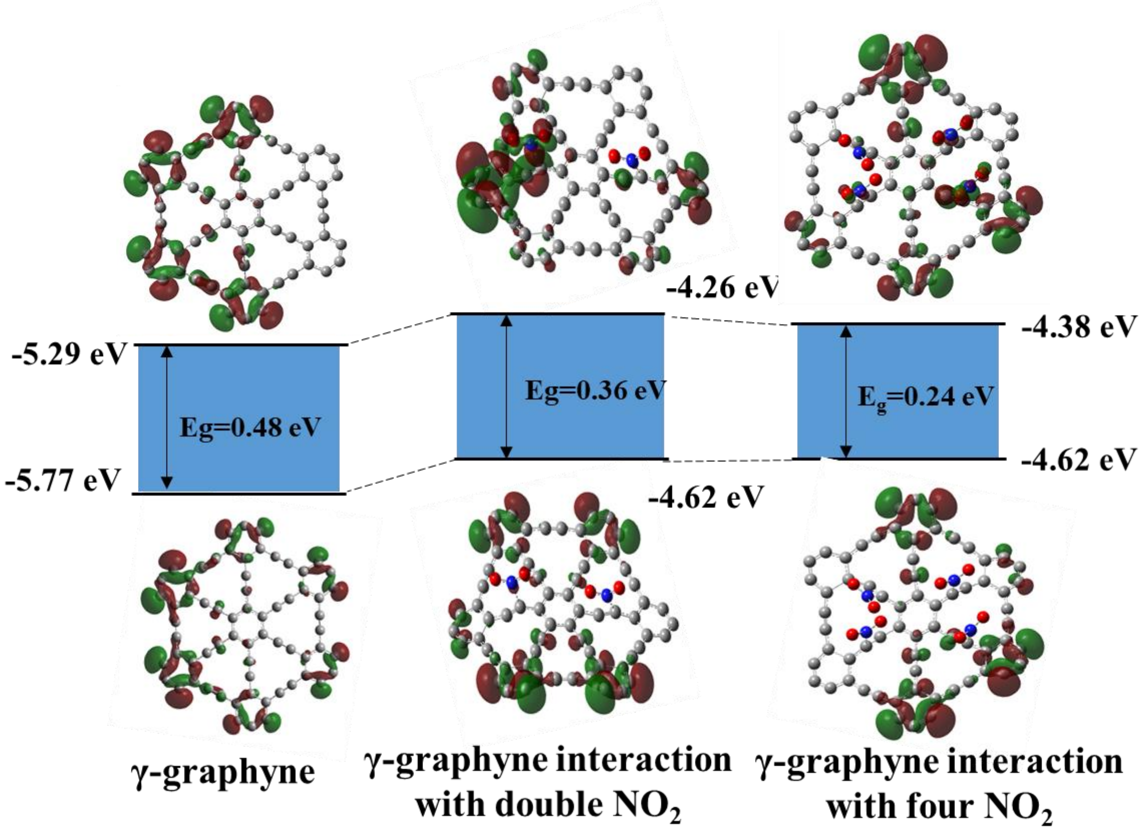

Fig. S5 Variation in HOMO and LUMO levels of graphyne after interaction with NO_2

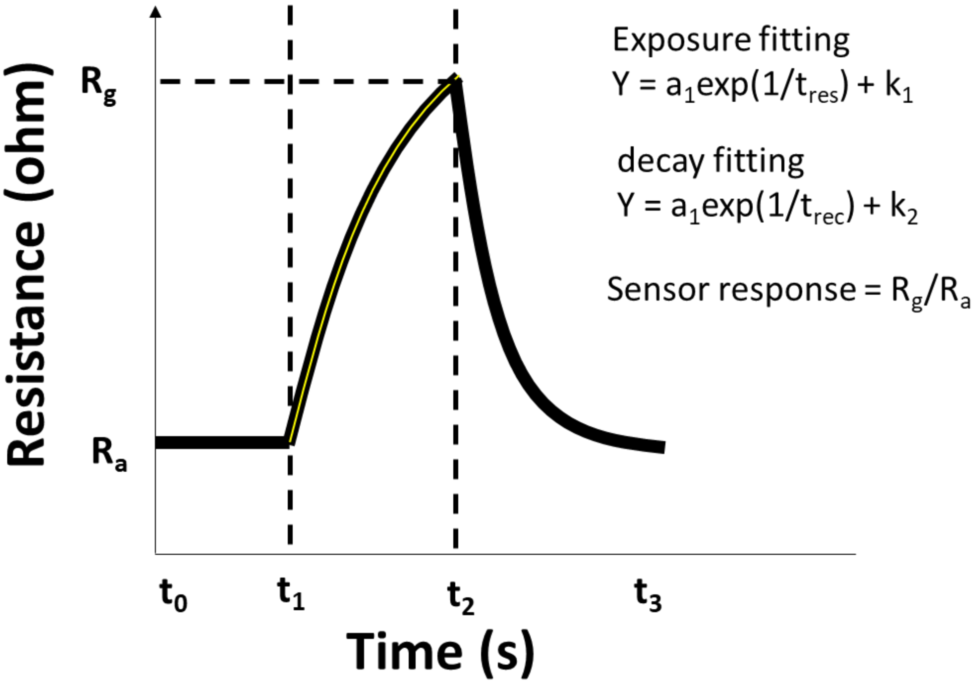

Fig. S6 Schematic sensing response for the single response profile
